# From tobacco smoking to cancer mutational signature: a mediation analysis strategy to explore the role of epigenetic changes 

**DOI:** 10.1186/s12885-020-07368-1

**Published:** 2020-09-14

**Authors:** Zhishan Chen, Wanqing Wen, Qiuyin Cai, Jirong Long, Ying Wang, Weiqiang Lin, Xiao-ou Shu, Wei Zheng, Xingyi Guo

**Affiliations:** 1 Division of Epidemiology, Department of Medicine, Vanderbilt-Ingram Cancer Center, Vanderbilt University Medical Center, Nashville, TN 37203 USA; 2 The Kidney Disease Center, the First Affiliated Hospital, Institute of Translational Medicine, Zhejiang University School of Medicine, Hangzhou, 310029 China; 3 Department of Biomedical Informatics, Vanderbilt University Medical Center, Nashville, TN 37203 USA

**Keywords:** Gene expression, Methylation, Tobacco smoking, Mutational signature, Mediation analysis

## Abstract

**Background:**

Tobacco smoking is associated with a unique mutational signature in the human cancer genome. It is unclear whether tobacco smoking-altered DNA methylations and gene expressions affect smoking-related mutational signature.

**Methods:**

We systematically analyzed the smoking-related DNA methylation sites reported from five previous casecontrol studies in peripheral blood cells to identify possible target genes. Using the mediation analysis approach, we evaluated whether the association of tobacco smoking with mutational signature is mediated through altered DNA methylation and expression of these target genes in lung adenocarcinoma tumor tissues.

**Results:**

Based on data obtained from 21,108 blood samples, we identified 374 smoking-related DNA methylation sites, annotated to 248 target genes. Using data from DNA methylations, gene expressions and smoking-related mutational signature generated from ~ 7700 tumor tissue samples across 26 cancer types from The Cancer Genome Atlas (TCGA), we found 11 of the 248 target genes whose expressions were associated with smoking-related mutational signature at a Bonferroni-correction *P* < 0.001. This included four for head and neck cancer, and seven for lung adenocarcinoma. In lung adenocarcinoma, our results showed that smoking increased the expression of three genes, *AHRR*, *GPR15*, and *HDGF*, and decreased the expression of two genes, *CAPN8*, and *RPS6KA1*, which were consequently associated with increased smoking-related mutational signature. Additional evidence showed that the elevated expression of *AHRR* and *GPR15* were associated with smoking-altered hypomethylations at cg14817490 and cg19859270, respectively, in lung adenocarcinoma tumor tissues. Lastly, we showed that decreased expression of *RPS6KA1*, were associated with poor survival of lung cancer patients.

**Conclusions:**

Our findings provide novel insight into the contributions of tobacco smoking to carcinogenesis through the underlying mechanisms of the elevated mutational signature by altered DNA methylations and gene expressions.

## Background

Tobacco smoking is a well-known risk factor for multiple cancer types, especially lung cancer [1–3]. DNA methylation, one of the major forms of epigenetic modification, essentially plays a regulatory role in gene expression. It has been a focus of multiple studies as a potential underlying molecular mechanism for tobacco smoking-related cancers. Previous epigenome-wide association studies (EWAS) have reported thousands of DNA methylations at CpG sites associated with tobacco smoking in blood, buccal cells and tumor-adjacent normal lung tissue samples [4–11]. These epidemiological studies have shown that tobacco smoking is consistently associated with DNA hypomethylated CpG sites in specific genes such as *AHRR* (encoding aryl-hydrocarbon receptor repressor) and *GPR15* (encoding G protein-coupled receptor 15) [12]. In particular, Stueve and colleagues identified seven smoking-associated hypomethylated CpG sites in adjacent normal tissues from 237 lung cancer patients. Of note, five of the seven sites, including a hypomethylated CpG site in *AHRR,* had been reported by previous blood-based EWAS, which suggests that methylation biomarkers identified from blood samples might reflect methylation changes in the target tissues [8].

Somatic mutations are one of the most common causes of carcinogenesis in humans [13, 14]. Recent studies using data from The Cancer Genome Atlas (TCGA) have created a landscape of somatic mutations in each cancer genome, ranging from hundreds to thousands of somatic mutations across multiple cancer types [14, 15]. To explore the biological processes of somatic mutations, Alexandrov and colleagues developed a mathematical framework to deconvolute them into mutational signatures. The approach characterized 96 mutation classifications that included six substitution types, together with a flanking base pair to the mutated base [15]. More than 30 mutational signatures have been identified across cancer types in TCGA [15, 16]. Previous studies have shown that a certain mutational signature was associated with tobacco smoking [15, 17, 18]. The smoking-related mutational signatures featured by predominantly C > A mutations with a transcriptional strand bias was observed in multiple human cancer types, including lung adenocarcinoma, lung small cell carcinomas, head and neck squamous, liver, larynx, oral cavity, and esophagus cancers [15, 17, 18]. Accumulating evidence has shown that dysregulated genes involved in DNA damage and repair could be responsible for mutational signature in the tumor genome [15, 17, 19, 20]. Examples of this are deficient mismatch repair (MMR), mutations in *POLE*, increased activity of the APOBEC family of cytidine deaminases, and DNA polymerase POLH [15, 16, 21]. Most recently, our own work has also shown that putative susceptibility genes may play a significant role in somatic mutations in human cancers [19]. Thus, we hypothesize that dysregulated genes, affected by tobacco smoking, may be also responsible for smoking-related mutational signatures in tumor tissues.

In our study, we evaluated the previously reported smoking-related DNA methylations from a total of 21,108 blood samples to identify candidate target genes [4–6, 10, 11]. Using data from DNA methylations, gene expressions and smoking-related mutational signature generated from approximately 7700 tumor tissue samples across 26 cancer types, we evaluated the associations of expression of these target genes with the smoking-related mutational signature in tumor tissues for each cancer type. Using a mediation approach, we further evaluated whether the association of tobacco smoking with the mutational signature may be mediated through an altered expression of these target genes in lung adenocarcinoma tumor tissues. Similar analyses were performed to evaluate the association of tobacco smoking with the gene expression mediated through smoking-altered DNA methylation.

## Methods

### Data resources

**Fig. 1:**
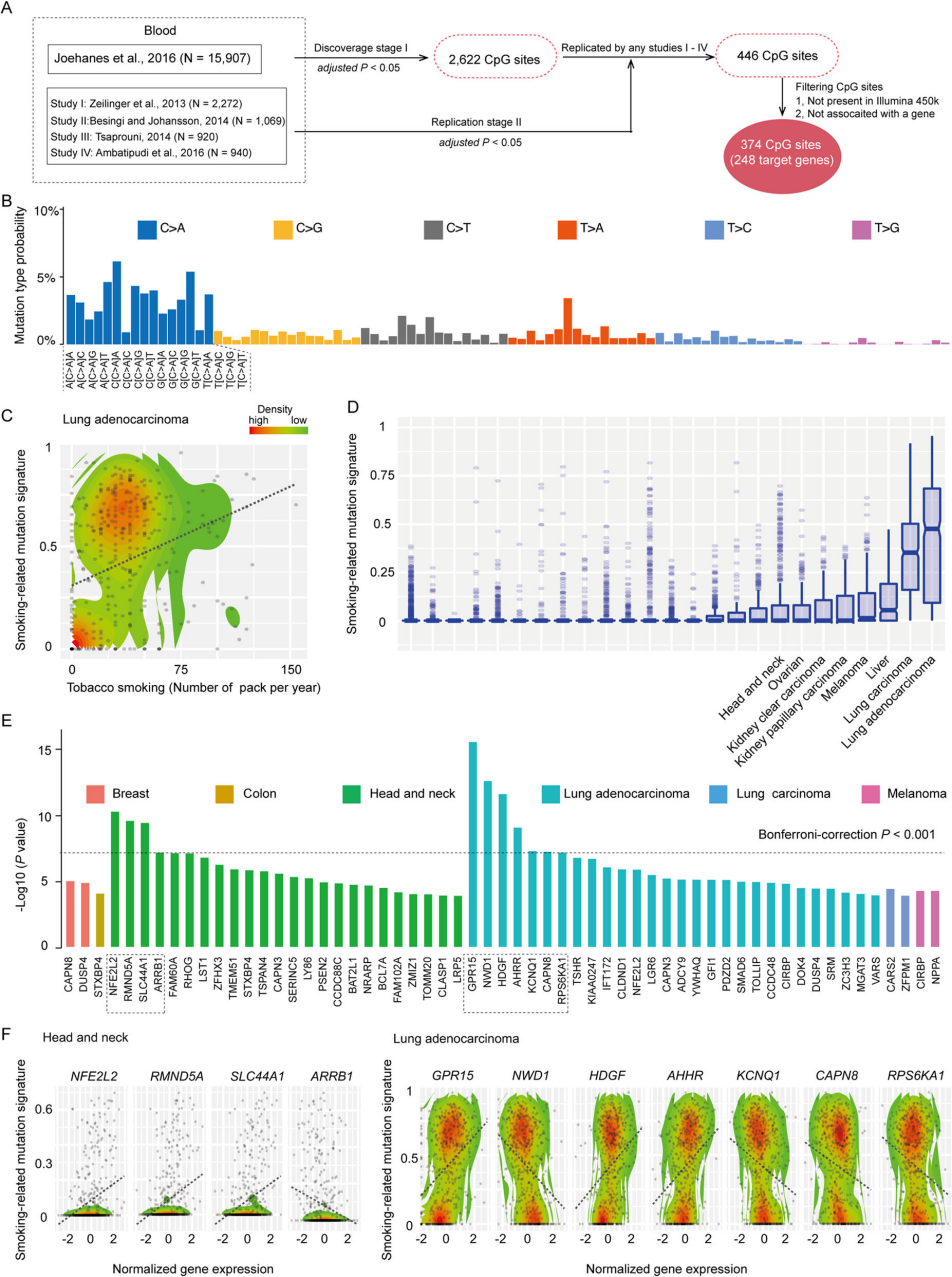
Identification of genes and their associations with smoking-related mutational signature. **a** A flow chart to illustrate the identification of candidate smoking-related DNA methylations from the previously reported blood-based methylations in five EWAS. “N” represents the sample size for each study. **b** Smoking-related mutational signature displayed according to the 96 substitution classifications characterized by six substitution types, together with a flanking base pair to the mutated base (Alexandrov et al. 2013). **c** A scatter plot indicating tobacco smoking correlated with known smoking-related mutational signature in lung adenocarcinoma. The dotted line refers to association coefficient. Each point represents one sample. The x axis represents the number of packs per year for each sample, the y axis represents the contribution of smoking-related mutational signature to overall mutation burden for each sample. The color from red to green refers to a higher to lower density of samples (this note applies to all other figure legends). **d** Box plots of the enrichment score of smoking-related mutational signature across 26 cancer types. **e** Bar plots indicating the *P* value of associations between the candidate genes and smoking-related mutational signature in six cancer types. Only genes with a *P* value of less than 1 × 10^− 4^ were presented. The dashed dot box highlights the genes with significant associations at a Bonferroni-correction *P* < 0.001. **f** Scatter plots for each gene with significant associations at a Bonferroni-correction *P* < 0.001. From the left to the right panel, four genes in head and neck and seven genes in lung adenocarcinoma are presented

We collected the previously reported smoking-related methylations in blood samples from five previous EWAS, including Joehanes et al., 2016 (*N* = 15,907) [6], Zeilinger et al., 2013 (*N* = 2272) [11], Besingi and Johansson, 2014 (*N* = 432) [5], Tsaprouni et al., 2014 (*N* = 920) [10], and Ambatipudi et al., 2016 (*N* = 940) [4]. All five of these studies included three categories of smoking status: current smoker, former smoker and never-smoker. We included the smoking-related methylations based on the comparison between current smoker and never-smoker. In the discovery stage, we only used the 2622 methylations at CpG sites reported from the study with the largest sample size (*N* = 15,907). In the replication stage, we only used methylations at CpG sites where we observed consistent associations in at least one other study at an adjusted *P* < 0.05 (Fig. 1). For the two EWAS studies from Zeilinger et al., 2012 and Tsaprouni et al., 2014 that were designed with both discovery and replication stages, only the CpG sites reported by both stages were used to replicate the findings from Joehanes et al., 2016 [6] in our analysis. We annotated methylation sites to their target genes based on the annotation from the Bioconductor package FDb.InfiniumMethylation.hg19 (version 2.2.0).

This study utilized multiple dimension datasets, including matched gene expression, DNA methylation, and clinical data that included age, gender and tobacco smoking. This was generated from 7757 samples in 26 cancer types from TCGA. The sample size for each cancer type is summarized in Supplementary Table 1. All the data were downloaded from TCGA using the Broad Institute Genome Data Analysis Center (GDAC) Firehose portal (stamp data/analyses__2016_01_28) through Firebrowse. Detailed information about datasets, analyses, and data sources are described at Firebrowse (http://gdac.broadinstitute.org/).

For gene expressions, the normalized expression levels for genes in tumor tissue samples were measured by RNA-Seq by Expectation Maximization (RSEM). To create a better distribution for downstream analysis, a log2 transfer of the RSEM values was applied. We used the Robust Multichip Average (RMA) approach to normalize the gene expression data across samples and to generate the same distribution for each sample. Furthermore, we transformed expression values for each gene across samples by an rank-based inverse normal transformation method for the downstream association analysis.

For DNA methylation, the data (Level 3) from the Illumina Infinium HumanMethylation450 BeadChip array for each sample in TCGA was measured. The Beta value of the methylation levels of each of the methylation sites were transformed to M value based on the equation $\mathrm{M}={\log}_2\left(\frac{Beta}{1- Beta}\right)$, using the function beta2m from the bioconductor package lumi (version 2.32.0) for the downstream analysis.

A total of 30 somatic mutational signatures for each sample in TCGA have been characterized from mSignatureDB (http://tardis.cgu.edu.tw/msignaturedb). We downloaded the data and only analyzed the known tobacco-associated “mutational signature 4” reported in the mSignatureDB, corresponding to tobacco-associated mutational signature in this study. We measured the enrichment score of this mutational signature for each sample (details described in our previous work [19]).

For gene expression microarray data of 541 lung adenocarcinoma patients, we downloaded the raw CEL files of four datasets (GSE30219, GSE31210, GSE37745 and GSE50081) from the Gene Expression Omnibus (GEO). These datasets with clinical survival information were screened out in a previous study [22]. The microarray data were processed using the RMA method from R package *affy*. The probes were mapped to genes using the annotation file of platform GPL570. The normalized expressions of probe set were aggregated into an expression level of the corresponding gene. The array batch effects were removed with the combat function from R package *sva*.

### The analysis of predicted neoantigen load

We downloaded the number of neoantigen loads for each sample from TCIA and applied log2 transfer to fit it into a better distribution. Mutational neoantigens were predicted by the use of HLA typing and MHC class I/II binding capabilities. The established neoantigen prediction algorithm NetMHCcons [23] was applied to missense somatic mutations to estimate their binding affinity to the HLA alleles. A more detailed analysis of the processing has been described in previous literature [24, 25].

### Statistical analysis

The distribution for relative contribution of smoking-related mutational signature to overall mutation burden is severely right-skewed. To better fit regression models, we used the ordinal semi-parametric regression models [26] to evaluate the associations of smoking-related mutational signature with tobacco smoking, gene expression and DNA methylation. Tobacco smoking variable was measured by smoking packs per year. The analyses were implemented in the ‘orm’ function from the ‘rms’ library of the R package [26]. To explore the mediation effects of DNA methylation on the association of tobacco smoking with smoking-related gene expression and the mediation effects of the smoking-related gene expression on the association of tobacco smoking with the smoking-related mutational signature, we conducted mediation analyses using the R package ‘mediation’ [27] to estimate the average direct effect (ADE) and the average causal mediation effect (ACME) of the mediators, which represent the population averages of these causal mediation and direct effects. A quasi-Bayesian approximation was used to construct their 95% confidence intervals. All the analyses were adjusted for age and gender. To estimate the association between the smoking-related gene expression and overall survival of lung cancer patients, we conducted survival analysis using the Cox proportional hazards model with the adjustment of age, gender and clinical stage.

## Results

### Identifying DNA methylations associated with tobacco smoking in blood samples

To identify smoking-related DNA methylations at CpG sites, we evaluated previously reported methylations in blood samples from five EWAS, including Joehanes et al., 2016 (*N* = 15,907), Zeilinger et al., 2013 (*N* = 2272), Besingi and Johansson, 2014 (*N* = 432), Tsaprouni, 2014 (*N* = 920), and Ambatipudi et al., 2016 (*N* = 940) (Fig. 1a) [4–6, 10, 11]. For our discovery data, we used a total of 2622 methylations at CpG sites reported by Joehanes et al’s study, which had the largest sample size. In the replication stage, we kept only those methylations at CpG sites which showed consistent associations in at least one of the remaining four studies (at the significance level of either Bonferroni or FDR adjusted *P* < 0.05 or genome-wide threshold of significance of *P* < 5 × 10^− 8^ in each EWAS) (Supplementary Table 2; see Methods). In the end, we identified a total of 374 smoking-related DNA methylations at CpG sites, annotated to 248 target genes (Fig. 1a; Supplementary Table 3). Of the 374 DNA methylations, the majority were hypomethylated CpG sites (*n* = 252, 67.4%), compared to hypermethylated CpG sites (*n* = 122, 32.6%).

### Identifying genes associated with smoking-related mutational signature in tumor tissues from a pan-cancer study

The smoking-related mutational signature was characterized in TCGA samples in previous studies [15, 28] (Fig. 1b). Utilizing this study, we used the relative contribution of the mutational signature to overall mutation burden, with values ranging from 0 to 1, for each sample across 26 cancer types in TCGA (see Methods). Using regression analyses, adjusting for gender and age, we observed that tobacco smoking was significantly associated with increased smoking-related mutational signature in lung adenocarcinoma (*P* = 1.75 × 10^− 9^; Fig. 1c). In line with previous studies, we observed that the contributions of smoking-related mutational signature to the overall mutation burdens varied in different cancers, with the most enrichments being observed in lung adenocarcinoma (median of contribution: 42%) and lung carcinoma (median of contribution: 35%) (Fig. 1d). Using regression analyses, adjusting for gender and age (see Methods), we evaluated the associations between the expressions of the identified 248 smoking-related target genes and smoking-related mutational signature for each cancer type. Of these target genes, we found that 234 genes were associated with smoking-related mutational signature in 19 cancer types (at a nominal *P* < 0.05) (Supplementary Table 4). At a more strict threshold of a *P* <  1 × 10^− 4^, a total of 59 genes were identified in six cancer types: breast (*n* = 2), colon (*n* = 1), head and neck (*n* = 24), lung adenocarcinoma (*n* = 28), lung carcinoma (n = 2), and melanoma (n = 2) (Fig. 1e; Supplementary Table 4).

In the end, we identified four genes for head and neck cancer and seven genes for lung adenocarcinoma, using a Bonferroni correction of *P* < 0.001 (alpha = 0.001 given 20,000 tests; *P* < 5 × 10^− 8^). Specifically, for head and neck cancer, the expression levels of three genes, *NFE2L2, RMND5A* and *SLC44A1*, were associated with increased smoking-related mutational signature, while an inverse association was observed for one gene, *ARRB1* (Fig. 1, Table 1). For lung adenocarcinoma, we found that the expression levels of three genes, *GPR15, HDGF,* and *AHHR*, were associated with increased smoking-related mutational signature, while an inverse association was observed for the other four genes, *NWD1, KCNQ1, CAPN8 and RPS6KA1* (Fig. 1, Table 1). *GPR15* showed the most significant association with a *P* < 2.22 × 10^− 16^ (Table 1).

**Table 1:** Associations between smoking-associated mutational signature and expression of candidate genes (Bonferroni-correction *P* < 0.01)

Cancer type	Gene	Beta	***P***
head and neck (*N* = 495)	*NFE2L2*	0.54	4.1 × 10^−11^
	*RMND5A*	0.56	2.0 × 10^−10^
	*SLC44A1*	0.56	2.9 × 10^−10^
	*ARRB1*	−0.46	5.1 × 10^− 8^
	*FAM60A*	0.44	5.8 × 10^− 8^
	*RHOG*	−0.43	5.9 × 10^− 8^
lung adenocarcinoma (*N* = 507)	*GPR15*	0.44	2.2 × 10^− 16^
	*NWD1*	−0.40	2.0 × 10^− 13^
	*HDGF*	0.42	1.9 × 10^− 12^
	*AHRR*	0.34	6.6 × 10^−10^
	*KCNQ1*	−0.29	3.9 × 10^− 8^
	*CAPN8*	−0.27	4.4 × 10^− 8^
	*RPS6KA1*	− 0.30	5.0 × 10^− 8^

“N” refers to sample size for each cancer type. A regression analysis was constructed to include tobacco smoking-associated mutational signature as a dependent variable and gene expression levels as the independent variable for each gene of each cancer type

### Mediation effects of the identified seven genes on the association of smoking with mutational signature in lung adenocarcinoma tumor tissues

**Fig. 2:**
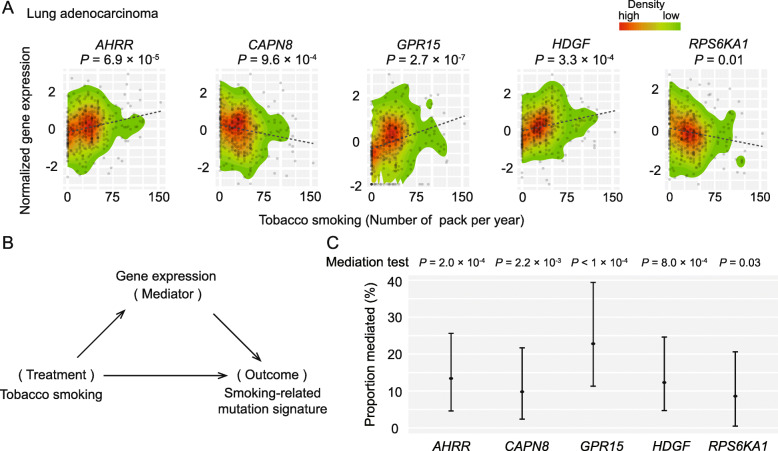
Mediation analysis illustrating the effect of the expression of five genes that would be altered by smoking on smoking-related mutational signature in lung adenocarcinoma. **a** Scatter plots indicating the statistical significance between five candidate genes and tobacco smoking in lung adenocarcinoma. **b** A diagram to illustrate a mediation analysis framework, where gene expression can be a mediator to affect smoking-related mutational signature. **c** Five candidate genes are presented with significant mediation effect (via gene expression on smoking-related mutational signature), at *P* < 0.05

For the identified seven genes for lung adenocarcinoma, we evaluated the associations between their expression and tobacco smoking (see Methods). We found that tobacco smoking was significantly associated with an increased expression of *AHRR*, *GPR15* and *HDGF* with a *P* = 6.9 × 10^− 5^, *P* = 2.7 × 10^− 7^ and *P* = 3.3 × 10^− 4^, respectively, and a decreased expression of *CAPN8* and *RPS6KA1* with a *P* = 9.6 × 10^− 4^ and *P* = 0.01, respectively (Fig. 2a; Supplementary Table 5). Notably, the associations of *AHRR*, *GPR15*, *HDGF* and *CAPN8* still reached a Bonferroni correction at *P* < 0.05 (given seven tests; *P* < 7.1 × 10^− 3^). Using a mediation analysis approach, we further estimated the ACME of the expression of these five genes that would be altered by smoking on the mutational signature. We found that they showed significant mediation effects on the association of smoking with the signature (Fig. 2c). Specifically, we observed a significant percentage of ACME for the smoking-related gene expressions: 13.4% (95% CI: 0.046 and 0.256) with a *P* = 2.0 × 10^− 4^ for *AHRR*, 9.8% (95% CI: 2.4 and 21.7%) with a *P* = 2.2 × 10^− 3^ for *CAPN8*, 22.8% (95% CI: 11.3 and 39.4%) with a *P* <  1 × 10^− 4^ for *GPR15*, 12.3% (95% CI: 4.7 and 24.6%) with a *P* = 8.0 × 10^− 4^ for *HDGF*, and 8.6% (95% CI: 0.5 and 20.6%) with a *P* = 0.032 for *RPS6KA1* (Fig. 2c; Table 2). Notably, the associations of *AHRR*, *CAPN8*, *GPR15* and *HDGF* still reached a Bonferroni correction at *P* < 0.05 (given five tests; *P* < 0.01).

**Table 2:** The direct effects of tobacco smoking, as well as the causal mediation (indirect) effects via gene expression, on the mutational signature in lung adenocarcinoma (*P* < 0.05)

Gene	Effect ^a^	Beta	95% CI		***P***
			Lower	Upper	
*AHRR*	ACME	4.5 × 10^− 4^	1.6 × 10^− 4^	8.3 × 10^− 4^	< 1.0 × 10^− 4^
	ADE	2.9 × 10^− 3^	1.7 × 10^− 3^	4.1 × 10^− 3^	< 1.0 × 10^− 4^
	Total Effect	3.3 × 10^− 3^	2.1 × 10^− 3^	4.5 × 10^− 3^	< 1.0 × 10^− 4^
	Prop	13.4%	4.6%	25.6%	2.0 × 10^− 4^
*CAPN8*	ACME	3.4 × 10^− 4^	8.2 × 10^− 5^	6.8 × 10^− 4^	< 1.0 × 10^− 4^
	ADE	3.0 × 10^− 3^	1.8 × 10^− 3^	4.2 × 10^− 3^	< 1.0 × 10^− 4^
	Total Effect	3.3 × 10^− 3^	2.1 × 10^− 3^	4.5 × 10^− 3^	< 1.0 × 10^− 4^
	Prop	9.8%	2.4%	21.7%	2.2 × 10^− 3^
*GPR15*	ACME	7.7 × 10^− 4^	3.9 × 10^− 4^	1.2 × 10^− 3^	< 1.0 × 10^− 4^
	ADE	2.6 × 10^− 3^	1.4 × 10^− 3^	3.7 × 10^− 3^	< 1.0 × 10^− 4^
	Total Effect	3.4 × 10^− 3^	2.2 × 10^− 3^	4.4 × 10^− 3^	< 1.0 × 10^− 4^
	Prop	22.8%	11.3%	39.4%	< 1.0 × 10^− 4^
*HDGF*	ACME	4.2 × 10^− 4^	1.6 × 10^− 4^	7.6 × 10^− 4^	< 1.0 × 10^− 4^
	ADE	2.9 × 10^− 3^	1.8 × 10^− 3^	4.1 × 10^− 3^	< 1.0 × 10^− 4^
	Total Effect	3.4 × 10^− 3^	2.2 × 10^− 3^	4.5 × 10^− 3^	< 1.0 × 10^− 4^
	Prop	12.3%	4.7%	24.6%	8.0 × 10^− 4^
*RPS6KA1*	ACME	3.0 × 10^− 4^	1.8 × 10^− 5^	6.7 × 10^− 4^	0.040
	ADE	3.0 × 10^− 3^	1.9 × 10^− 3^	4.2 × 10^− 3^	< 1.0 × 10^− 4^
	Total Effect	3.3 × 10^− 3^	2.1 × 10^− 3^	4.5 × 10^− 3^	< 1.0 × 10^− 4^
	Prop	8.6%	5%	20.6%	0.032

“^a^”: “ACME” refers to the average causal mediation effects. “ADE” refers to the average direct effects. “Prop” refers to the proportion of the total effect of tobacco smoking on the mutational signature mediated by the gene expression

### Mediation effects of smoking-related DNA methylation on the association of smoking with gene expression in lung adenocarcinoma tumor tissues

**Fig. 3:**
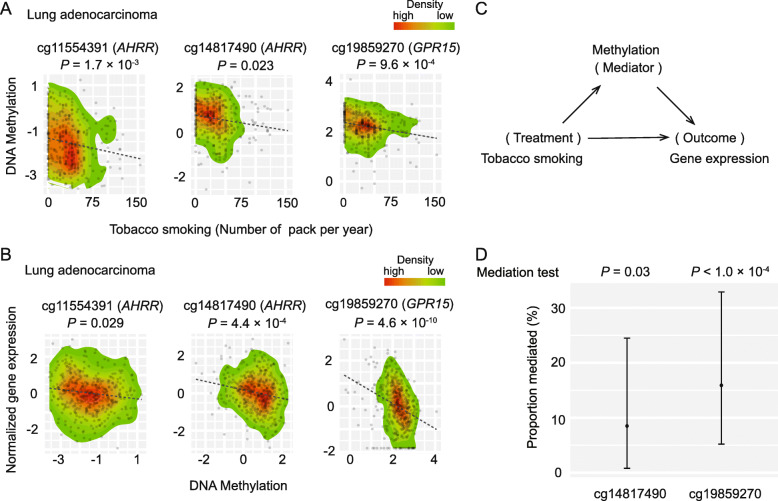
Mediation analysis illustrating the effect of tobacco smoking-altered methylation on gene expression in lung adenocarcinoma. **a** Scatter plots indicating the statistical significance of associations between methylations at three candidate CpG sites and tobacco smoking in lung adenocarcinoma. **b** Scatter plots indicating negative correlations between DNA methylation at three candidate CpG sites and gene expression in lung adenocarcinoma.**c** A diagram to illustrate a mediation analysis framework, where DNA methylation can be a mediator to affect the expression of tobacco smoking-altered genes. **d** Two candidate CpG sites are presented with significant mediation effects on gene expression, at *P* < 0.05. “ACME” refers to the average causal mediation effects via DNA methylation on gene expression

In the above mediation analysis, we found that five genes, *AHRR*, *CAPN8*, *GPR15*, *HDGF*, and *RPS6KA1,* mediated the association between smoking and mutational signature in lung adenocarcinoma. For these, six smoking-related DNA methylations, cg11554391, cg14817490, cg21446172, cg19859270, cg00867472 and cg13092108, have been reported in blood cells [4–6, 10, 11]. We further evaluated the associations between these methylations and tobacco smoking in lung adenocarcinoma tumor tissues. In line with previous findings from case-control studies of blood samples, we found that consumed tobacco smoke was significantly associated with hypomethylations at the CpG sites cg11554391 (*AHRR*), cg14817490 (*AHRR)*, and cg19859270 (*GPR15)* in lung cancer tumor tissues (*P* < 0.05 for all; Fig. 3a; Supplementary Table 5). The associations of cg11554391 (*AHRR)*, and cg19859270 (*GPR15)* still reached a Bonferroni correction at *P* < 0.05 (given six tests; *P* < 0.008). Next, we evaluated the association between the methylation at each CpG site and gene expression. Interestingly, our results showed that the smoking-altered hypomethylations at cg11554391 and cg14817490 were associated with an elevated expression of *AHRR*; the smoking-altered hypomethylation at cg19859270 was associated with an elevated expression of *GPR15* (*P* < 0.05 for all), indicating that these smoking-altered hypomethylations likely play an up-regulation role in their gene expression (Fig. 3b; Supplementary Table 6). Notably, the associations for cg14817490 (*AHRR)* and cg19859270 (*GPR15)* still reached a Bonferroni correction at *P* < 0.05 (given six tests; *P* < 0.008). In particular, these hypomethylated CpG sites are located in regions with evidence of enhancer activities associated with their target genes (Supplementary Figure 1). In addition, we also analyzed the associations between a total of seven isoforms of *AHRR* and DNA methylations at CpG sites in lung adenocarcinoma tumor tissues (Supplementary Table 7). In line with the above observation, we observed that three majorly expressed isoforms of *AHRR*, uc003jaw, uc003jay and uc003jaz, were negatively associated with DNA methylation at cg11554391 (Supplementary Table 6). These isoforms are also negatively associated with methylation cg14817490, while only the isoform uc003jaw showed statistical significance (Supplementary Table 6). No significant associations were observed for the remaining isoforms due to their low expression, indicating our analysis in the gene level may only reflect the major expressed isoforms (Supplementary Figure 2). Similarly, we observed that the isoforms of *GPR15*, uc001apq and uc010oad, were negatively associated with the DNA methylation at cg19859270 (Supplementary Table 6).

Using a mediation analysis approach, we further estimated the ACME of the methylations that would be altered by smoking on gene expressions. We found that the methylations at two CpG sites, *AHRR* (cg14817490, *P* = 0.03) and *GPR15* (cg19859270, *P* <  1 × 10^− 4^), showed significant mediation effects on the association of smoking with gene expression (Fig. 3c, d; Table 3). Specifically, we observed a significant percentage of ACME for both smoking-related DNA methylations: 8.5% (95% CI: 8 and 24.5%) with a *P* = 0.03 for *AHRR*, and 15.9% (95% CI: 5.2 and 32.9%) with a *P* <  1.0 × 10^− 4^ for *GRP15* (Fig. 3d; Table 3).

**Table 3:** The direct effects of tobacco smoking, as well as the causal mediation (indirect) effects via DNA methylation, on the gene expression in lung adenocarcinoma (*P* < 0.05)

CpG	Effect ^a^	Beta	95% CI		***P***
			Lower	Upper	
cg14817490 (*AHRR*)	ACME	6.5 × 10^−4^	5.7 × 10^−5^	1.5 × 10^−3^	0.03
	ADE	6.5 × 10^−3^	3.1 × 10^− 3^	1.0 × 10^− 2^	< 1.0 × 10^− 4^
	Total Effect	7.2 × 10^− 3^	3.8 × 10^− 3^	1.1 × 10^− 2^	< 1.0 × 10^− 4^
	Prop	8.5%	8%	24.5%	0.03
cg19859270 (*GPR15*)	ACME	1.5 × 10^− 3^	4.6 × 10^− 4^	2.9 × 10^− 3^	< 1.0 × 10^− 4^
	ADE	7.8 × 10^− 3^	4.4 × 10^− 3^	1.1 × 10^− 2^	< 1.0 × 10^− 4^
	Total Effect	9.3 × 10^− 3^	5.8 × 10^− 3^	1.3 × 10^− 2^	< 1.0 × 10^− 4^
	Prop	15.9%	5.2%	32.9%	< 1.0 × 10^−4^

^a^ ACME refers to the average causal mediation effects. ADE refers to the average direct effects (ADE). “Prop” refers to the proportion of the total effect of tobacco smoking on the gene expression mediated by DNA methylation

### Overall survival analysis for *AHRR*, *CAPN8*, *GPR15*, *HDGF and RPS6KA* in lung cancer adenocarcinoma

To explore the association between overall survival of lung cancer patients and the identified five genes that mediated the association between smoking and mutational signature in lung adenocarcinoma, we conducted the Cox regression analysis using data from TCGA (see Methods). Our results revealed that the elevated expression level of *RPS6KA1* was associated with the increased overall survival of lung cancer patients, when comparing the high level of gene expression (>median) to low level (<=median) (Hazard Ratio [HR] = 0.64, *P* = 5.9 × 10^− 3^) (Supplementary Table 8). This association was further evaluated using public data (*n* = 541 lung cancer patients; see Methods). We showed that the elevated expression level of *RPS6KA1* was consistently associated with the increased overall survival of lung cancer patients with HR = 0.78, and a marginal significance of *P* = 0.09. These findings are in line our initial results that tobacco smoking decreased expression level of *RPS6KA1*. No significant associations with overall survival of lung cancer patients were observed for other four genes.

## Discussion

In the present study, a total of 374 smoking-related methylations annotated to 248 target genes were identified using strict statistical criteria from previous EWASs in blood samples. Using data from TCGA, we identified a total of 11 candidate genes of 248 target genes whose expressions were associated with smoking-related mutational signature, including four in head and neck cancer and seven in lung adenocarcinoma. Of seven genes for lung adenocarcinoma, our results further showed that smoking increased the expression of three genes, *AHRR*, *GPR15*, and *HDGF*, and decreased the expression of two genes, *CAPN8*, and *RPS6KA1*. These smoking-altered gene expressions were consequently associated with increased smoking-related mutational signature. In addition, our results showed that the elevated expressions of *AHRR* and *GPR15* were associated with smoking-altered hypomethylations of cg14817490 and cg19859270 in both lung cancer blood and tumor tissues, respectively.

Our analysis focused on the identified 374 blood-based methylations associated with tobacco smoking, which have strong evidence of statistical associations from previous studies. In particular, the initial discovery of methylations associated with tobacco smoking is based on a study with the largest sample size we have found so far (*N* = 15,907) (see Methods) [6]. In addition to studies of blood, two studies have investigated methylations associated with tobacco smoking in buccal cells (*N* = 790) [9] and tumor adjacent normal lung tissue (*N* = 237) [8]. Notably, both studies had limited sample sizes and were insufficient in statistical power to identify smoking-related methylation sites, while they have revealed evidence that blood-based methylation biomarkers could reflect changes in their target tissues. Recently, Ma and Li performed pathway enrichment analyses based on 320 smoking-affected genes identified in blood. Their results showed that 104 of these genes were significantly enriched in pathways associated with the etiology of different cancers [29]. Consistent with these findings, two recent epidemiology studies showed that smoking-related hypomethylations in blood cells were associated with lung cancer risk [30, 31]. Thus, our study shows a connection of blood-based methylations with tobacco smoking-related mutational signature in tumor tissue. It should be noted that other confounders such as body mass index (BMI) and alcohol consumption data are not available for lung adenocarcinomas in TCGA, which prevents us from including these variables as confounders. Nevertheless, we provided statistical evidence that tobacco smoking leading to carcinogenesis through the underlying mechanisms of the elevated mutational signature that was likely mediated by altered DNA methylations and gene expressions.

Using the median analysis, we evaluated associations of smoking-related DNA methylations and gene expressions with the smoking-related mutational signature in lung adenocarcinoma. Thus, the identified dysregulated genes that were likely affected by tobacco smoking, may contribute to generating the smoking-related mutational signature in lung adenocarcinoma. Notably, the smoking variable of pack years was used for our association analysis. In addition, we evaluated the association smoking status (smoker and non-smoker) with between both gene expressions and DNA methylations at CpG sites in lung adenocarcinoma*.* Overall, we showed that associations based on smoking status were consistently associated with the results using smoking represented by smoking packs per year, while the latter variable as a continuous variable could slightly increase statistical power (Supplementary Table 5). Previous studies have suggested that the *AHRR* gene was associated with tobacco smoking, based on EWAS from blood, buccal cell and normal lung tissue [4–11]. In recent studies, the hypomethylated CpG sites in the *AHRR* gene in pre-diagnostic peripheral blood samples were reported to be associated with lung cancer risk [30, 31]. Based on in vitro experiments from both humans and mice, the evaluated *AHRR* expression has been validated by tobacco smoking-altered methylations [7]. However, the *AHRR* is a putative tumor suppressor gene encoding a competitive suppressor of the aryl hydrocarbon receptor (AHR). The *AHRR - AHR* negative feedback loop plays an essential role in detoxifying dioxin, including polycyclic aromatic hydrocarbons (PAHs), an important class of smoking carcinogens [32, 33]. In addition to *AHRR*, *GPR15* encodes an orphan G-protein-coupled receptor involved in the regulation of innate immunity and T-cell trafficking in the intestinal epithelium [34, 35]. Similarly, the biological mechanisms of how *GPR15* contribute to smoking-related mutational signatures in lung adenocarcinoma remain unclear. Nevertheless, we provided candidate genes that significantly contributed to smoking-related mutational signature in lung cancer. Further functional characterization for these genes needs to be conducted to provide biological evidence and explore oncogenic pathways for their effects on smoking-related mutational signature.

Our results showed three additional genes, *CAPN8*, *HDGF* and *RPS6KA1,* may be involved in smoking-related mutational signature, mediated by gene expression altered by tobacco smoking in lung adenocarcinoma. Tobacco smoking-related methylations in these genes have been reported in the previous EWAS in blood samples. However, we did not observe that these methylations were associated with tobacco smoking in lung adenocarcinoma, although consistent association directions were observed for *HDG* and *RPS6KA1* (Data not shown). Notably, unlike the studies in large sample size from blood studies, the statistical analysis in detecting association between DNA methylation and tobacco smoking is still challenge in tumor tissues due to possible factors, such as tumor heterogeneity, potential confounders, and limited sample size. In fact, our focus on the analysis of the reported blood-based smoking-related DNA methylation sites could identify reliably smoking-related target genes and reduce the possibility of reverse causation. Nevertheless, given the tissue-specificities of some methylations in blood, further studies with a large sample size are still needed to replicate the associations for these candidate tobacco smoking-related genes in lung adenocarcinoma. In fact, our results showed that smoking-related methylations of these genes were associated with decreased expressions of these genes (*P* < 0.01 for all), indicating that they may play a down-regulation role in their gene expression in lung adenocarcinoma (Supplementary Figure 3). Further in vitro or in vivo functional assays are needed to validate the genes that are affected by tobacco smoking in lung cancer.

It is known that neoantigens (or neoepitopes) result from missense somatic mutations in cancer cells [36]. However, how smoking-related mutational signature contribute to neoantigen loads remain unclear. We additionally evaluated the associations between smoking-related mutation signature and predicted neoantigen loads (see Methods). We observed that smoking-related mutational signature were significantly associated with increased neoantigen loads in three cancer types, head and neck, lung adenocarcinoma, and lung carcinoma (see Methods). An inverse association was observed in melanoma (*P* <  1 × 10^− 4^ for all; Supplementary Figure 4A, B; Supplementary Table 9). The most significant association was observed in lung adenocarcinoma with a *P* < 2.2 × 10^− 16^. In addition, we also observed that neoantigen loads were associated with all five identified genes (*P* <  1 × 10^− 5^) and tobacco smoking (*P* = 2.16 × 10^− 11^) in lung adenocarcinoma (Supplementary Figure 4C, D). In particular, the expressions of *AHRR* and *GPR15* had associations with an increased predicted neoantigen load with *P* = 7.6 × 10^− 10^ and *P* = 7.7 × 10^− 7^, respectively (Supplementary Figure 4D). Thus, our findings may provide new clues to explore the biological and immunological mechanisms through which smoking-related mutational signature may be involved in carcinogenesis, and provide potential genomic biomarkers for the development of cancer prevention and immunotherapy.

## Conclusions

Our results showed that the smoking-altered DNA methylations and gene expressions play an important role in contributing to smoking-related mutational signature in human cancers. Our results also indicated that tobacco-smoking plays an important role in clinical significance, likely affecting genes with the impact on overall survival of lung cancer patients. Our study not only provides candidate genes that contribute to tobacco smoking carcinogenesis, but also can potentially lead to a new avenue for target intervention.

## Supplementary information

**Additional file 1: Table S1.** The sample size for each cancer from TCGA. **Table S2.** A collection of candidate blood-based methylations at CpG sites reported from five previous epigenome wide association studies. **Table S3.** A list of 374 candidate blood-based methylation CpG sites and genes identified from both discovery and replication studies at an adjusted *P* < 0.05. **Table S4.** Associations between smoking-associated mutational signature and expression of candidate genes for each cancer type (*P* < 0.05). **Table S5.** Associations between tobacco smoking and expression of candidate genes and as methylation of candidate CpG sites. **Table S6.** Association of expression of candidate genes and their isoforms with methylation at each CpG site. **Table S7.** Correlation between expressions of *AHRR* and its isoforms. **Table S8.** Cox regression analysis between gene expression and overall survival in lung cancer patients. **Table S9.** Associations between smoking-associated mutational signature and predicted neoantigen load for each cancer type.

**Additional file 2:** **Figure S1.** The epigenetic landscape of regions with methylations at two candidate CpG sites.

**Additional file 3:** **Figure S2.** Boxplots showing the expression of *AHRR* and its isoforms in lung adenocarcinoma tumor tissues (*n* = 512).

**Additional file 4: Figure S3.** Associations between gene expressions and methylations at three CpG sites.

**Additional file 5:**: **Figure S4.** Smoking-related mutational signature contributed to neoantigen load in multiple cancer types.

## Data Availability

The normalized expressions of gene and DNA methylation were downloaded from the TCGA using the Broad Institute Genome Data Analysis Center (GDAC) Firehose portal through Firebrowse (stamp data/analyses__2016_01_28, http://gdac.broadinstitute.org). Somatic mutational signatures were downloaded from mSignatureDB (http://tardis.cgu.edu.tw/msignaturedb). Neoantigen data was downloaded from TCIA (https://tcia.at/home).

## Abbreviations

AHRR: Aryl-Hydrocarbon Receptor Repressor
CAPN8: Calpain 8
EWAS: Epigenome-Wide Association Studies
GPR15: G Protein-Coupled Receptor 15
HDGF: Heparin Binding Growth Factor
HR: Hazard Ratio
RPS6KA1: Ribosomal Protein S6 Kinase A1
TCGA: The Cancer Genome Atlas
