# Supplementary figures and images for: From tobacco smoking to cancer mutational signature: a mediation analysis strategy to explore the role of epigenetic changes
Source: BMC Cancer. 2020 Sep 14;20:880. doi: 10.1186/s12885-020-07368-1 (PMC7488848; doi:10.1186/s12885-020-07368-1)

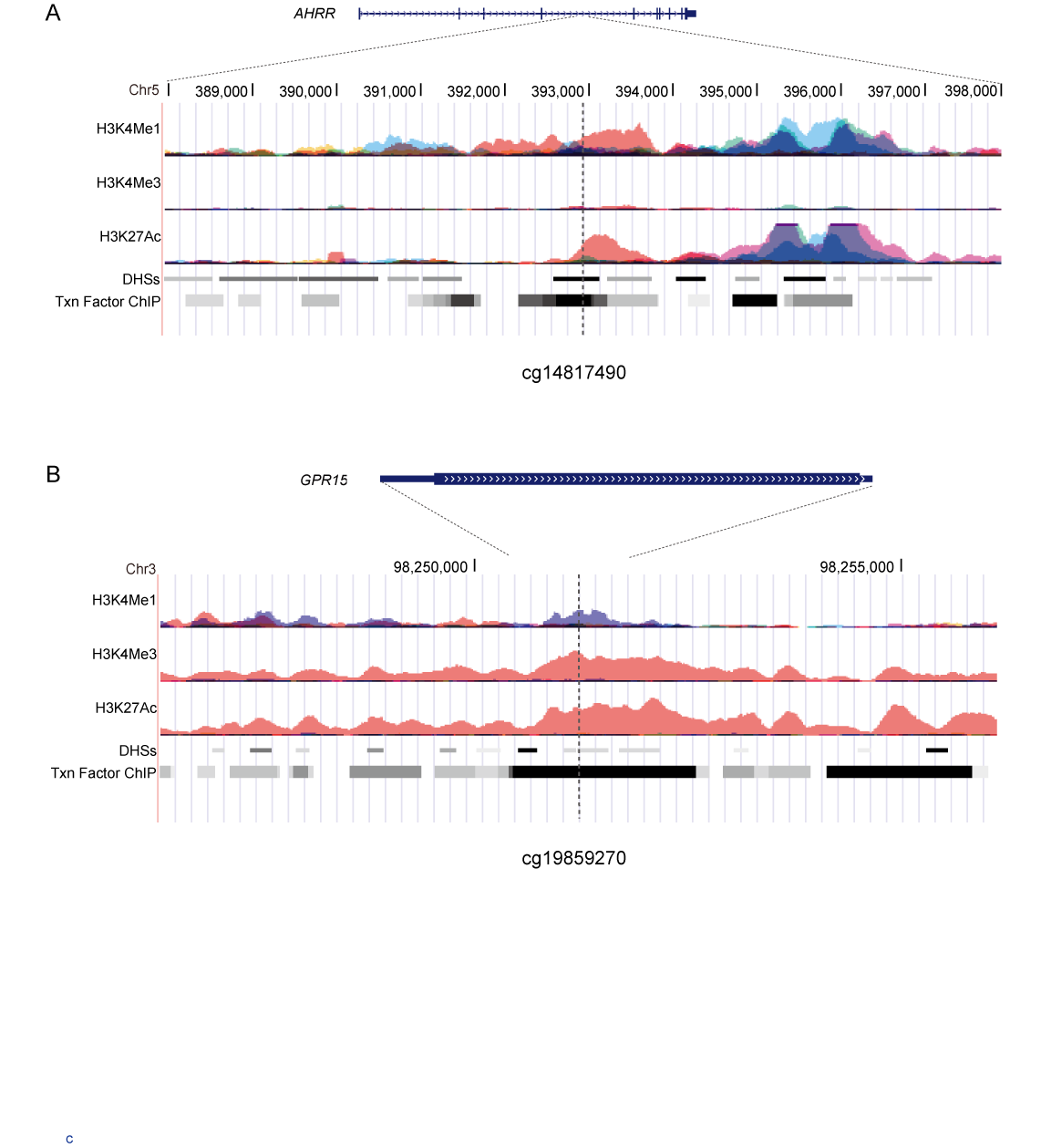

Supplement: Supplementary file 2 — Additional file 2: Figure S1. The epigenetic landscape of regions with methylations at two candidate CpG sites. [file 12885_2020_7368_MOESM2_ESM.tif]

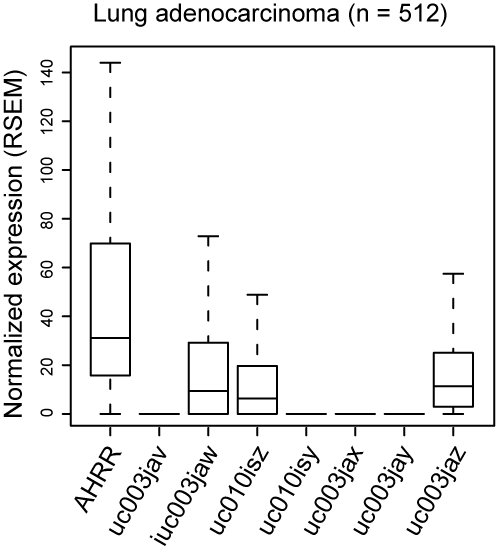

Supplement: Supplementary file 3 — Additional file 3: Figure S2. Boxplots showing the expression of AHRR and its isoforms in lung adenocarcinoma tumor tissues (n = 512). [file 12885_2020_7368_MOESM3_ESM.tif]

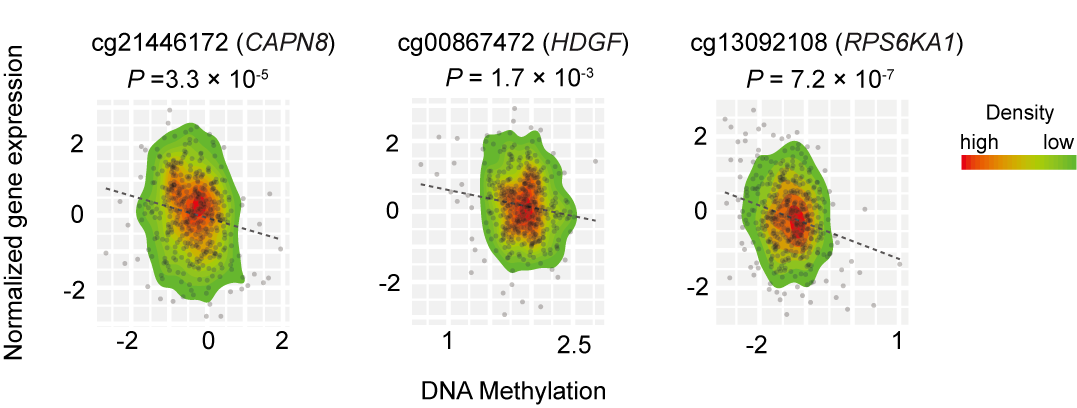

Supplement: Supplementary file 4 — Additional file 4: Figure S3. Associations between gene expressions and methylations at three CpG sites. [file 12885_2020_7368_MOESM4_ESM.tif]

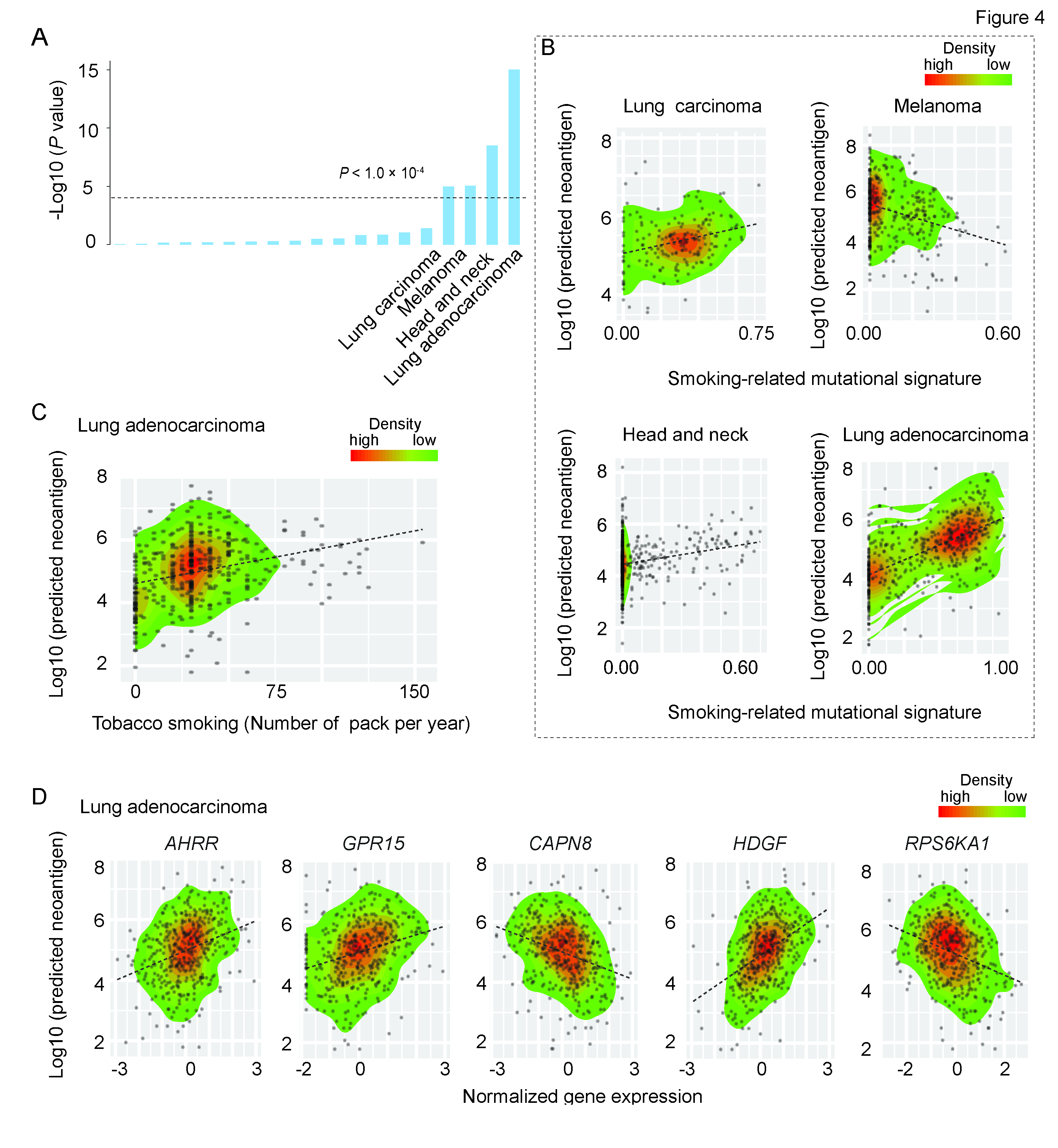

Supplement: Supplementary file 5 — Additional file 5:: Figure S4. Smoking-related mutational signature contributed to neoantigen load in multiple cancer types. [file 12885_2020_7368_MOESM5_ESM.tif]
